# Treatment of Kimura disease with dupilumab: a case report and literature review

**DOI:** 10.3389/fimmu.2026.1670489

**Published:** 2026-02-16

**Authors:** Wei Yang, Ze Wu, Jun Niu

**Affiliations:** Department of Dermatology, General Hospital of Northern Theater Command, Shenyang, China

**Keywords:** biologics, case report, corticosteroid, Kimura disease, management

## Abstract

Kimura disease is a benign chronic inflammatory condition characterized by painless subcutaneous masses, peripheral blood eosinophilia, and increased serum immunoglobulin E levels. The pathogenesis of KD remains unclear, however, more and more evidence suggests that type 2 inflammation may play an important role in KD. We present a case of a 19-year-old male who complained of masses behind both ears for two months, ultimately diagnosed as KD. He showed a partial response to the initial systemic corticosteroid therapy. However, after tapering the dosage of corticosteroids, the patient’s symptoms did not improve, even with combined mycophenolate mofetil therapy. His symptoms worsened significantly after he discontinued treatment. Therefore, treatment with dupilumab was initiated at 600 mg, followed by 300 mg every two weeks. At week 20, the treatment was adjusted to 300 mg every four weeks for a duration of 32 weeks. The mass gradually reduced in size, accompanied by a progressive decrease in both the eosinophil count and IgE levels. These results show the significant efficacy of dupilumab in the management of KD.

## 1 Introduction

Kimura disease (KD) is an infrequent benign chronic inflammatory disorder with an unknown cause. The typical presentation of KD includes painless subcutaneous masses in the head and neck region, regional lymphadenopathy, elevated eosinophil (EO) counts in blood, and increased levels of immunoglobulin E (IgE) (1, 2). Histopathologic features include lymphoid follicular hyperplasia, eosinophil infiltration, angioproliferation, and perilesional fibrosis (3). The current therapeutic options mainly include surgery, corticosteroids, immunotherapy, and radiation (4, 5). The pathogenesis of KD is complicated and poorly understood. In recent years, with the development of biological agents, new treatment approaches for KD have emerged. In this report, we present a case of KD that was treated with dupilumab.

## 2 Case description

**Figure 1:**
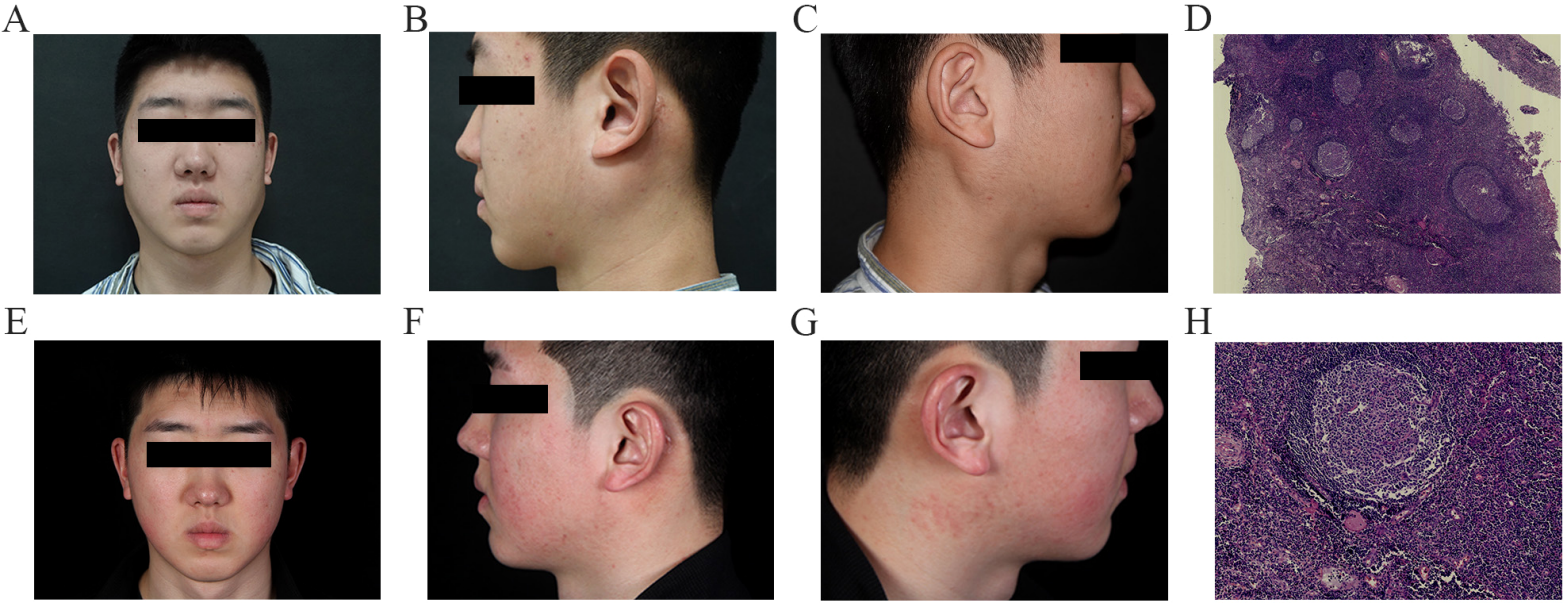
Clinical symptoms observed in the patient before treatment included bilateral parotid gland enlargement and palpable masses behind both ears **(A–C)**. After 52 weeks of dupilumab therapy, bilateral parotid gland swelling decreased significantly, and the masses behind both ears became noticeably smaller **(E–G)**. The lymph node biopsy **(D, H)** revealed numerous lymphatic follicles with germinal centers. Infiltration of lymphocytes, plasma cells, eosinophils, and swelling vascular endothelial cells were noted.

**Figure 2:**
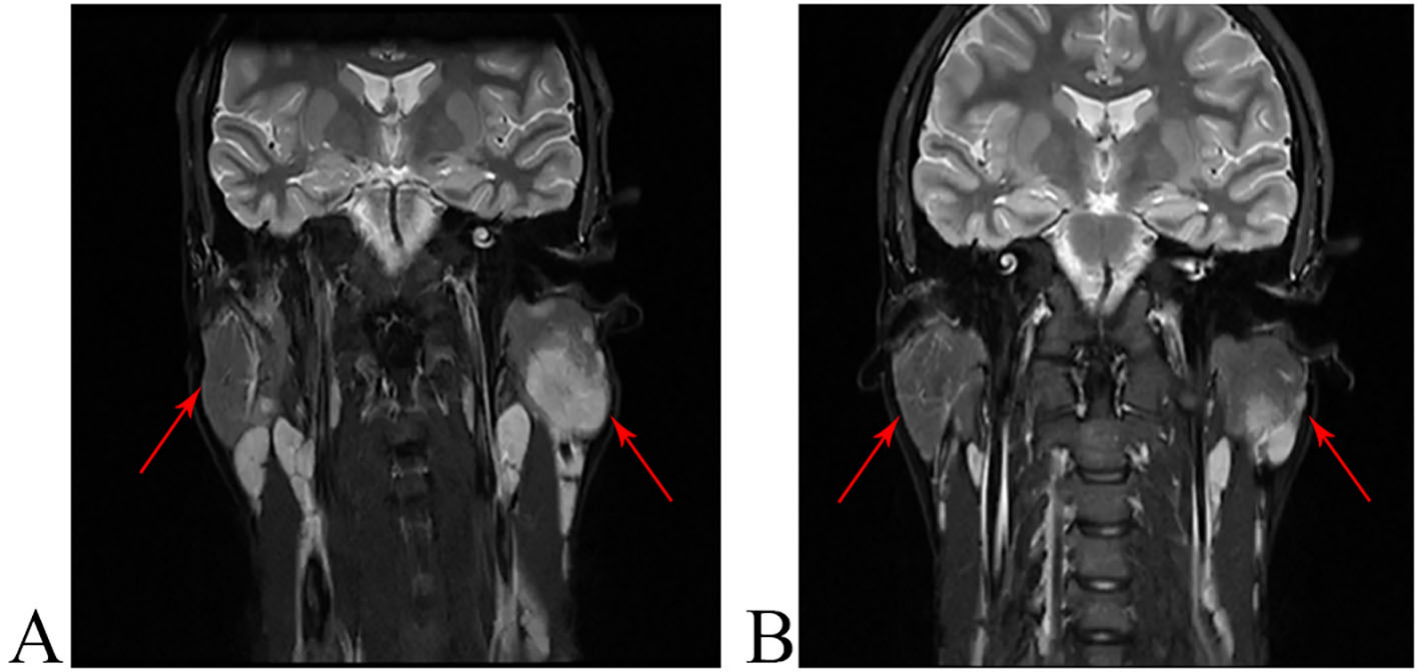
Radiological appearance of KD lesions on short-tau inversion-recovery T2-weighted fast spin-echo images. **(A)** The MRI results before treatment with dupilumab showed multiple space-occupying lesions in both parotid glands, with the largest measuring 2.8×2.3×3.5cm. **(B)** At week 20 of treatment, the MRI still showed multiple space-occupying lesions in both parotid glands, but the largest one measured only 1.8 × 0.6 × 2.4 cm.

A 19-year-old male complained of masses behind both ears for two months. Intravenous cefuroxime and ribavirin were administered for two weeks at a local hospital, however, the masses gradually increased in size. A biopsy was conducted, and the result indicated eosinophilic lymphatic granuloma. Subsequently, he was admitted to our hospital. Admission blood tests indicated the following: white blood cell (WBC) count 11.2×10^3^/µL (normal 3.5 - 9.5×10^3^/µL), EO count 3.57×10^3^/µL (normal 0.02 - 0.52×10^3^/µL), IgE 1080IU/ml (normal < 100 IU/ml). The results for hepatic, renal functions, and urinalysis were within normal limits. The bone marrow biopsy suggested eosinophilia. Ultrasound revealed enlarged lymph nodes in the neck, parotid glands, and supraclavicular region. According to the clinical and histopathological findings, a diagnosis of Kimura’s disease was made. Oral methyl-prednisolone (32mg/d) was administered. Two weeks after the treatment, the cervical, supraclavicular, and parotid lymph nodes regressed, the size of the masses slightly decreased, the absolute EO count reduced to 1.74×10^3^/µL, but the IgE level rose to 1550 IU/ml. The methyl-prednisolone dose was reduced to 16mg, and mycophenolate mofetil (1.0g/d) was added. This treatment lasted for four months, however, there was no significant change in the masses. Due to personal reasons, medication was discontinued, and the masses began to enlarge again. Upon his return to our department, he had been off medication for 10 months. The size of the masses was larger than when he first visited our department (**Figures 1A–C**). Laboratory results indicated a WBC count of 14.3×10^3^/µL, an EO count of 7.06×10^3^/µL, and an IgE level of 4810 IU/ml. The MRI results indicate multiple space-occupying lesions in both parotid glands, with the largest measuring 2.8×2.3×3.5cm (**Figure 2A**). Treatment with dupilumab was initiated at 600mg, followed by 300mg every two weeks. At the 16-week follow-up, blood tests revealed a WBC count of 10.7×10^3^/µL, an EO count of 1.41×10^3^/µL, and an IgE level of 1180 IU/ml. At week 20 of treatment, we performed a follow-up MRI, which still showed multiple space-occupying lesions in both parotid glands, but the largest one measured only 1.8×0.6×2.4cm (**Figure 2B**). Subsequently, the treatment was adjusted to 300mg every four weeks for a duration of 32 weeks (**Figures 1E–G**). Now the treatment has been adjusted to 300mg every six weeks, and the condition has been well controlled.

## 3 Discussion

KD is a benign lymphoproliferative disorder that is more prevalent in Asian male populations (1). The pathogenesis of KD has not been fully clarified, however, it may be closely associated with immunological factors. Patients with KD expressed high levels of interleukin (IL)-4, IL-5, and IL-13 messenger RNA in peripheral blood mononuclear cells (6). The numbers of peripheral blood Th2 and T cytotoxic type 1 cells increase significantly, accompanied by elevated levels of IL-4, IL-5, IL-10, and IL-13 (7, 8). IL-4 can induce B-cell class switching to IgE antibodies, resulting in further enhancing the immune response (9, 10). These factors may account for the significant elevation in both eosinophil counts and serum IgE levels observed in KD patients. Besides, type 2 immune cells are abundant in tissue lesions of KD (11). Dupilumab, which blocks IL-4 and IL-13 signaling, shows good efficacy in treating KD (12). These findings suggest that type 2 inflammation may play an important role in the pathogenesis of KD.

The main differential diagnoses for KD include angiolymphoid hyperplasia with eosinophilia (ALHE) and other conditions associated with peripheral lymphadenopathy. ALHE typically presents as superficial skin or subcutaneous nodules, accompanied by normal eosinophils or mild eosinophilia and the absence of increased IgE, and it is histologically characterized by a predominant vascular component and by an inflammatory infiltrate (13, 14). The other differential diagnoses include lymphoma, leukemia, tuberculosis, and so on. Therefore, we performed a bone marrow aspiration and lymph node biopsy on the patient. The bone marrow showed eosinophilia. The lymph node biopsy revealed numerous lymphatic follicles with germinal centers. Infiltration of lymphocytes, plasma cells, and swelling vascular endothelial cells were noted (**Figures 1D, H**).

**Figure 3:**
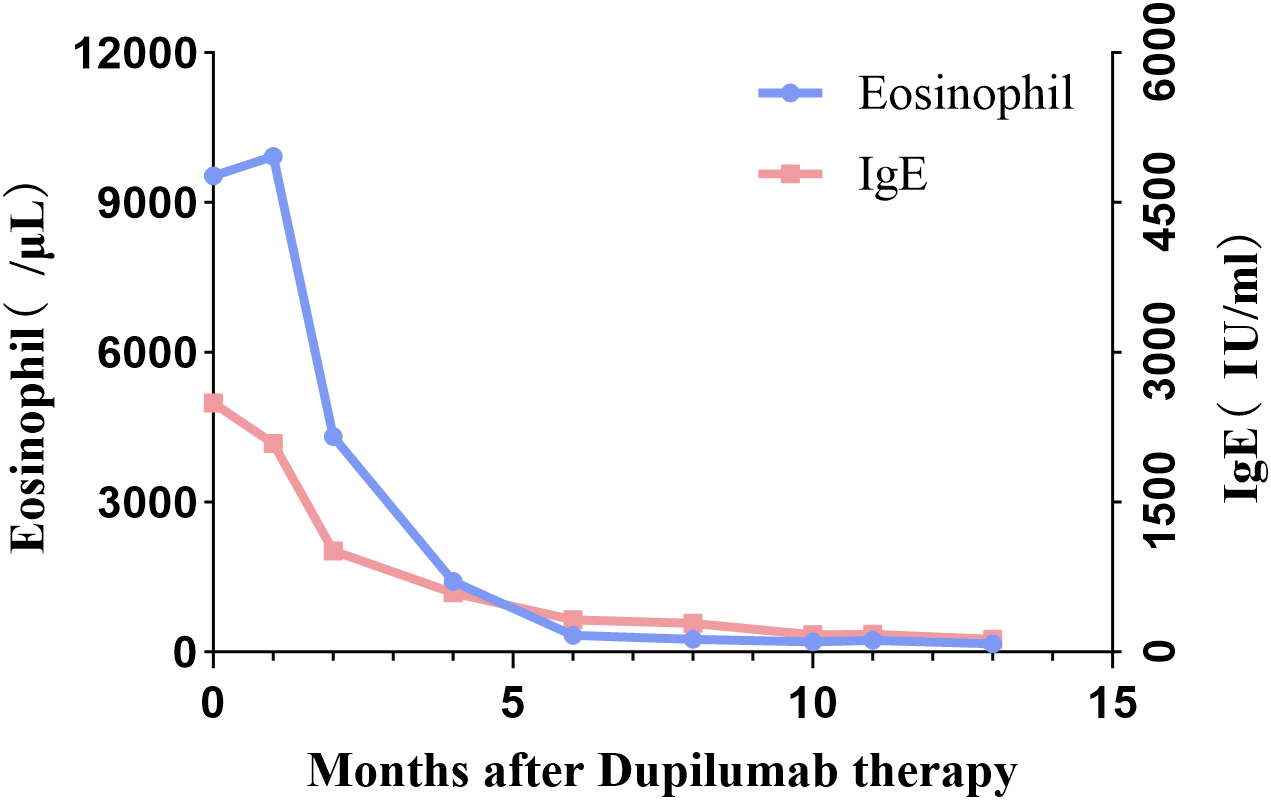
Time-course changes in the levels of eosinophil counts and serum IgE in the patient.

The management of KD remains challenging due to the absence of a standardized treatment. Most of the clinical evidence is provided by case series and case reports. Surgical excision may be considered for localized lesions, particularly when presenting as solitary masses (7). However, due to the infiltrative growth of the lesions, poorly defined margins, multifocal nature of the disease, and potential involvement of local lymph nodes, complete resection is challenging. This may result in a high recurrence rate (15). Corticosteroids therapy typically achieves favorable responses. However, this treatment shows some limitations, including high relapse rates during dose tapering or discontinuation, immune suppression, osteoporosis, and other adverse effects (12). Radiation therapy may be an appropriate option for recurrent cases or poor surgical candidates. However, a wide range of radiotherapy doses has been administered to patients with KD, but an optimal dosage has not yet to be established (4, 16). Additionally, there may be risks associated with radiation exposure and potential toxicity. As for our patient, systemic corticosteroids were used for initial treatment. The size of the mass had decreased. However, after the reduction of the corticosteroid dosage, the variation in size became rather inconspicuous, even with the concomitant administration of mycophenolate mofetil. Moreover, upon the patient discontinuing his medications by himself, the mass enlarged again, and its size even exceeded that before the corticosteroid treatment. In this condition, dupilumab was given. The mass gradually reduced in size, accompanied by a progressive decrease in both the eosinophil count and IgE levels. Similarly, previous studies showed the efficacy of dupilumab in treating KD with most treatment regimens mirroring those used for atopic dermatitis (1, 7, 12, 17–24). As with most reported cases of KD treated with dupilumab, our initial therapeutic approach also followed the standard dosing protocol established for atopic dermatitis (**Table 1**). After 20 weeks of standard induction therapy at 300 mg biweekly, the patient achieved partial remission. While cases have reported symptom remission with dupilumab in KD, few have investigated dose reduction strategies or long-term maintenance therapy. Notably, among those cases that have attempted extended dosing intervals, no disease recurrence was observed (12, 21). In this context, considering both the substantial financial burden and the marked therapeutic response, we decided to extend the administration interval to 300 mg every four weeks, which resulted in continued clinical remission. Encouraged by this sustained response, we further extended the interval to 300mg every six weeks. Notably, the patient has maintained excellent disease control on this six-weekly regimen for 12 months, with no recurrence of postauricular masses, sustained normalization of eosinophil counts, and progressive decline in IgE levels (**Figure 3**). However, the evidence level is limited, and further research is required to evaluate dose reduction strategies.

**Table 1:** Summary of reported cases of Kimura disease treated with dupilumab.

Reference	Ethnicity	Age	Sex	Duration	Combination therapy	Dupilumab dose	treatment interval	Initial evaluation time	Dupilumab treatment duration	Follow-up duration	Outcome
Luo SY et al. (1)	Asian	25y	F	6m	–	600 mg/300mg	2w	1m	–	7m	CR
Huang HY et al. (7)	Asian	36y	M	2y	Surgery	600 mg/300mg	2w	4m	8m	16m	CR
Teraki Y et al. (12)	Asian	57y	M	10y	–	600 mg/300mg	2w/4w	4m	–	10m	PR
Shang BS et al. (17)	Asian	14y	M	2y	Corticosteroid	600 mg/300mg	2w	2m	10m	10m	PR
Cordeil S et al. (18)	African	77y	M	22y	–	600 mg/300mg	2w	–	12m	12m	CR
Lyu Y et al. (19)	Asian	37y	F	3y	Corticosteroid	600 mg/300mg	2w	4m	4m	9m	PR
Bellinato F et al. (20)	African	59y	M	20y	–	300mg	2w	2m	–	6m	PR
Liu YL et al. (21)	Asian	10y	M	8y	Corticosteroid	600 mg/300mg	2w	3m	6m	6m	CR
	Asian	35y	M	3.5y	Corticosteroid Thalidomide	600 mg/300mg	2w	3m	6m	6m	CR
	Asian	11y	M	0.3y	Corticosteroid	600 mg/300mg	2w	3m	6m	6m	CR
	Asian	36y	M	13y	Corticosteroid	600 mg/300mg	2w	3m	6m	6m	PR
	Asian	11y	M	11y	Corticosteroid	600 mg/300mg	2w	3m	6m	6m	CR
	Asian	44y	M	33y	Corticosteroid Methotrexate	600 mg/300mg	2w	3m	6m	6m	PR
Suga K et al. (22)	Asian	65y	M	2y	–	600 mg/300mg	2w	1m	15m	15m	PR
Battesti G et al. (23)	African	44y	M	1y	–	600 mg/300mg	2w	6m	12m	12m	CR
	African	46y	F	10y	–	600 mg/300mg	2w	6m	12m	12m	PR
Zheng Y et al. (24)	Asian	69y	M	6m	–	–	2w	6m	6m	6m	CR
Present Case	Asian	19y	M	2m	–	600mg/300mg	2 w/4w/6w	4m	24m	24m	CR

F, female; M, male; w, weeks; m, months; y, years; CR, complete remission; PR, partial remission.

In addition to dupilumab, other biological agents such as omalizumab, mepolizumab, benralizumab, and rituximab have shown potential in treating KD. Omalizumab, an anti - IgE monoclonal antibody, can directly bind to free IgE, inhibit its binding to the high-affinity receptor for IgE (FcϵRI) on the surface of mast cells and basophils, and suppress cell activation. Meanwhile, it can also bind to membrane-bound IgE on the surface of B cells, inhibit the synthesis of IgE by B cells, and prevent the occurrence of allergic reactions (25). Previous literature has shown that omalizumab can gradually reduce the count of eosinophils and make the lesions shrink and soften in patients with KD (26). However, there is also a report indicating that omalizumab treatment is ineffective (27). Therefore, we speculate that IgE is partly involved in the pathogenesis of KD. And the application prospects of omalizumab in the treatment of KD need to be explored. Moreover, in KD patients, mepolizumab treatment resulted in a reduction in mass size and eosinophil count within the tissue (28). Additionally, successful treatment of KD patients with benralizumab has also been reported (29). Anti-IL-5/IL-5Rα therapy can suppress eosinophil production and activity, while also reducing their recruitment and lifespan. Taken together, anti-IL-5/IL-5Rα therapy might be an effective approach by reducing eosinophils. Rituximab specifically binds to the CD20 B-cell antigen expressed on B lymphocytes, leading to the destruction of B lymphocytes and a reduction in antibody secretion. It can reduce nodular lesions, eliminate germinal centers, and alleviate B-cell infiltration in KD patients, while significantly lowering serum IgE levels (30). Furthermore, it is effective in KD patients with renal involvement (31). Therefore, rituximab may be an option for treating KD patients with renal involvement.

However, the use of biologics in treating KD is primarily based on case series studies or case reports. The long-term safety and efficacy of these treatments remain uncertain. Additionally, biologics may cause adverse effects, while most adverse effects are mild, they still require close monitoring.

In summary, the pathogenesis of KD seems to be closely related to the type II inflammatory response. Biologics, such as dupilumab, offer new therapeutic options for KD. Dupilumab works mainly by blocking the IL-4 and IL-13 signaling pathways and modulating immune cell functions. However, there are still limitations in the use of biologics for treating KD. Further study should focus on exploring the efficacy and safety of biologics in the treatment of KD and improving patient outcomes.

## Data Availability

The original contributions presented in the study are included in the article/supplementary material. Further inquiries can be directed to the corresponding author.
